# Comparative Gynecological Safety of the Dual GIP/GLP-1 Receptor Agonist Tirzepatide vs. the GLP-1 Receptor Agonist Semaglutide: A Real-World Pharmacovigilance Analysis (2022–2025)

**DOI:** 10.7759/cureus.101738

**Published:** 2026-01-17

**Authors:** Hima Bindu Makkena

**Affiliations:** 1 Internal Medicine, Independent Research, McKinney, USA

**Keywords:** faers, glp-1 receptor agonists, gynecological hemorrhage, pharmacovigilance, semaglutide, tirzepatide

## Abstract

Background

The rapid adoption of incretin-based therapies for obesity has raised questions regarding their comparative safety profiles. While the gynecological safety of glucagon-like peptide-1 (GLP-1) receptor agonists is documented, the potential risks associated with dual glucose-dependent insulinotropic polypeptide (GIP)/GLP-1 agonism remain less characterized. This study aimed to evaluate the reporting patterns of gynecological hemorrhagic events associated with tirzepatide compared to semaglutide using real-world pharmacovigilance data.

Methods

A retrospective disproportionality analysis was conducted of the FDA Adverse Event Reporting System (FAERS) database from Q1 2022 to Q3 2025. Cases of gynecological hemorrhage were identified using standardized MedDRA queries. Reporting odds ratios (RORs) with 95% confidence intervals (CIs) were calculated to compare the reporting frequency of hemorrhagic events between tirzepatide and semaglutide.

Results

A total of 103,607 female-specific reports were analyzed (tirzepatide: n = 70,768; semaglutide: n = 32,839). Gynecological hemorrhagic events were reported in 0.60% of tirzepatide cases and 0.62% of semaglutide cases. The ROR for tirzepatide versus semaglutide was 0.97 (95% CI = 0.82-1.14), indicating no statistically significant difference in reporting odds. A significant disparity in reporter sources was observed, with 94.6% of tirzepatide reports originating from consumers compared to 53.4% for semaglutide.

Conclusions

This analysis of post-marketing surveillance data did not detect a disproportionate reporting signal suggesting an increased risk of gynecological hemorrhagic events with tirzepatide compared to semaglutide. While these findings suggest a comparable safety profile, the high proportion of consumer-driven reporting for tirzepatide warrants cautious interpretation. Future prospective studies are needed to confirm these findings in controlled clinical settings.

## Introduction

Background

The therapeutic landscape for obesity and type 2 diabetes (T2D) has been fundamentally transformed by the widespread adoption of nutrient-stimulated hormone-based therapies. While glucagon-like peptide-1 (GLP-1) receptor agonists, such as semaglutide, established the efficacy of incretin-based weight management, the recent introduction of tirzepatide, a first-in-class dual glucose-dependent insulinotropic polypeptide (GIP) and GLP-1 receptor agonist, has set a new benchmark. Clinical trials, including the SURMOUNT-1 [1] and SURPASS-2 [2] studies, have demonstrated that tirzepatide can yield body weight reductions exceeding 20%, significantly surpassing outcomes typically seen with GLP-1 monotherapy [3,4]. As prescription volumes for these agents surge globally, characterizing their safety profile in real-world populations remains a critical pharmacovigilance priority.

The clinical gap

The safety profile of tirzepatide is currently well-characterized regarding gastrointestinal adverse events (nausea, vomiting, pancreatitis) and potential thyroid risks. However, a specific clinical question has emerged from post-marketing surveillance and patient communities: the impact of these potent agents on gynecological health. Anecdotal reports and preliminary broad-spectrum data mining have suggested a potential link between the initiation of these therapies and menstrual disturbances, ranging from irregular cycles to unexpected postmenopausal hemorrhage. Despite these concerns, no dedicated pharmacovigilance study has yet comprehensively compared the gynecological hemorrhagic risk of tirzepatide against the established standard of care, semaglutide, in a concurrent real-world cohort.

Biological plausibility

A potential mechanism linking these therapies to uterine bleeding involves the endocrine function of adipose tissue. Adipose tissue serves as a reservoir for endogenous sex steroids, particularly estrogens [5]. The rapid and profound lipolysis induced by high-potency agents such as tirzepatide may theoretically result in the acute release of sequestered estrogen into the systemic circulation (“estrogen dumping”). This transient hyperestrogenic state could stimulate endometrial proliferation and subsequent shedding. Given that tirzepatide induces greater weight loss than semaglutide, a hypothesis exists that it might carry a proportionately higher risk of such gynecological adverse events.

Study objective

By analyzing reports from the post-marketing period of 2022-2025, this study aimed to evaluate the reporting patterns of gynecological hemorrhagic events associated with the dual GIP/GLP-1 receptor agonist tirzepatide compared to the GLP-1 receptor agonist semaglutide. This study was designed as a signal-detection analysis to determine if the addition of GIP receptor agonism correlates with a disproportionate reporting signal, acknowledging the inherent limitations of spontaneous reporting systems in establishing causal incidence.

## Materials and methods

Data source

A retrospective pharmacovigilance study was conducted using data from the FDA Adverse Event Reporting System (FAERS) Public Dashboard and quarterly data files [6]. The study period was restricted to January 1, 2022, through September 30, 2025, to ensure a concurrent comparison period following the market approval of tirzepatide (approved May 2022). To minimize temporal bias, the comparator group (semaglutide) was restricted to the identical reporting period (Q1 2022 to Q3 2025).

Study population and case definition

The analysis was limited to female subjects. We identified cases where the “Primary Suspect” drug was either tirzepatide or semaglutide, encompassing both brand-name and compounded formulations to ensure comprehensive capture of real-world usage. Tirzepatide search terms included Mounjaro, Zepbound, tirzepatide (generic), and compounded formulations including cyanocobalamin/tirzepatide and cyanocobalamin/glycine/tirzepatide. Semaglutide search terms included Ozempic, Wegovy, Rybelsus, semaglutide (generic), and compounded formulations including cyanocobalamin/semaglutide. Data integrity was maintained by verifying unique Case IDs to ensure that multiple versions of the same case (e.g., follow-up reports) were not counted more than once.

Adverse event identification

Gynecological hemorrhagic events were identified using the Medical Dictionary for Regulatory Activities (MedDRA) terminology. A composite endpoint was created using the following Preferred Terms (PTs): Heavy Menstrual Bleeding, Postmenopausal Haemorrhage, Uterine Haemorrhage, Vaginal Haemorrhage, Genital Haemorrhage, Abnormal Uterine Bleeding, Polymenorrhoea, Intermenstrual Bleeding, and Menometrorrhagia.

Statistical analysis

Disproportionality analysis was conducted using the reporting odds ratio (ROR) [7,8]. The ROR compares the odds of a specific adverse event occurring with the target drug (tirzepatide) versus a comparator drug (semaglutide). Statistical significance was determined using the 95% confidence interval (CI). In pharmacovigilance studies, the 95% CI serves as the surrogate for hypothesis testing [9]; if the interval includes the null value of 1.0, the association is not statistically significant (p > 0.05). All data processing and statistical calculations were performed using Microsoft Excel (version 16.0).

## Results

Descriptive epidemiology

A total of 103,607 female-specific adverse event reports were included in the final analysis for the 2022-2025 study period. The tirzepatide cohort consisted of 70,768 reports, while the semaglutide comparator cohort consisted of 32,839 reports. Geographic distribution of cases varied, with the United States and Great Britain representing the primary reporting sources. Reporter demographics notably differed between cohorts: 94.6% of tirzepatide-associated bleeding cases (n = 403) were reported by consumers, compared to 53.4% (n = 109) in the semaglutide cohort.

Gynecological hemorrhagic events

We identified 426 cases of gynecological hemorrhage associated with tirzepatide, representing a proportional reporting rate of 0.60%. In the comparator group, semaglutide was associated with 204 cases, representing a reporting rate of 0.62%. Among the tirzepatide-associated bleeding events, 241 (56.6%) were classified as “Serious” adverse events. Similarly, in the semaglutide cohort, 121 (59.3%) cases were classified as “Serious.” In both cohorts, an analysis of outcome data indicated that the majority of these cases were classified as “Other Medically Significant” (events not requiring hospitalization but deemed clinically important), while confirmed hospitalizations represented a minority of the serious subset. There were zero reported deaths attributed to gynecological hemorrhage in either cohort.

Disproportionality analysis

The calculated ROR for gynecological hemorrhage with tirzepatide versus semaglutide was 0.97 (95% CI = 0.82-1.14). As the CI crossed 1.0, there was no statistically significant difference in the reporting probability of gynecological hemorrhagic events between tirzepatide and semaglutide. Demographic and baseline characteristics are summarized in Table 1, while comparative pharmacovigilance metrics are presented in Table 2.

**Table 1:** Demographic and baseline characteristics of the study population. Data are presented as N and n (%). *: Age statistics derived from the subset of bleeding cases with available age data (tirzepatide: n = 239; semaglutide: n = 124).

Metric	Tirzepatide study drug)	Semaglutide (comparator)
Total female reports, N	70,768	32,839
Age of cases (years)		
Mean ± SD*	46.0 ± 12.3	45.1 ± 13.5
Reporter type, n (%)		
Healthcare professional	23 (5.4%)	95 (46.6%)
Consumer/Patient	403 (94.6%)	109 (53.4%)
Primary reporter country, n (%)		
United States	195 (45.8%)	112 (54.9%)
Great Britain	195 (45.8%)	41 (20.1%)
Other/Unknown	36 (8.4%)	51 (25.0%)

**Table 2:** Comparative pharmacovigilance analysis of gynecological hemorrhage signals. Statistical analysis: Disproportionality analysis was performed using the ROR. Statistical significance is determined by the 95% CI; a result is considered statistically significant (p < 0.05) if the 95% CI entirely excludes the null value of 1.0. ROR = reporting odds ratio; CI = confidence interval

Metric	Tirzepatide (study drug)	Semaglutide (comparator)
Total bleeding cases (n)	426	204
Case outcomes, n (%)		
Serious cases	241 (56.6%)	121 (59.3%)
Fatal cases	0 (0.0%)	0 (0.0%)
Signal analysis		
Reporting rate (%)	0.60%	0.62%
ROR	0.97	Reference
95% CI	0.82–1.14	—
Statistical significance	Not significant (p > 0.05)	—

## Discussion

This study provides a comparative pharmacovigilance assessment of gynecological safety for tirzepatide versus semaglutide in a real-world setting. The results indicate that despite the novel mechanism of action and superior weight loss efficacy of tirzepatide, there is no evidence of an increased reporting signal for gynecological hemorrhagic events relative to the established GLP-1 agonist class.

The impact of reporting sources and social media trends

A notable finding in our analysis is the significant disparity in reporter type: 94.6% of tirzepatide-associated hemorrhagic events were consumer-reported, compared to 53.4% for semaglutide. This likely reflects the intense social media attention and “viral” status tirzepatide has achieved as a weight-loss agent during the study period. High consumer reporting often captures fewer medically verified events, potentially inflating the signal with minor or self-limiting bleeding episodes that a healthcare professional might not deem reportable. Conversely, the higher proportion of healthcare professional reports for semaglutide suggests a more clinical, medically adjudicated safety profile for the established comparator.

Clinical context and mechanisms

The relationship between rapid weight loss and menstrual irregularities is well-documented. Adipose tissue functions as a significant endocrine organ, sequestering steroid hormones [5]. We hypothesized that the rapid lipolysis induced by tirzepatide, which demonstrated superior weight reduction compared to semaglutide in the SURMOUNT-1 trial [1], might result in a “dumping” of stored estrogen into systemic circulation. However, our findings indicate that this theoretical risk does not translate into a disproportionately elevated rate of adverse event reporting. This may imply that the “GIP” component of tirzepatide does not exert an additive deleterious effect on the hypothalamic-pituitary-gonadal (HPG) axis [10]. Alternatively, it is possible that the gynecological side effects of incretin-based therapies are a class effect driven primarily by metabolic shifts rather than direct receptor interaction in the reproductive tract [11].

Clinical interpretation: adverse event versus physiological restoration

It is critical to contextualize “bleeding” within the metabolic effects of these drugs. Many women prescribed incretin-based therapies suffer from obesity-related anovulation or polycystic ovary syndrome (PCOS). Significant weight loss is known to improve insulin sensitivity and restore the HPG axis, often leading to the resumption of spontaneous menses in previously amenorrheic patients. Therefore, a subset of these reported “hemorrhagic events” may clinically represent the restoration of normal physiological function (the “PCOS Paradox”) rather than direct drug toxicity. Additionally, the delayed gastric emptying associated with both GIP and GLP-1 agonists may alter the absorption kinetics of oral contraceptives, which could serve as a confounding factor for breakthrough bleeding.

Signal detection and compounded formulations

It is important to note that FAERS data reflect reporting patterns rather than true clinical incidence. The presence of compounded formulations in the marketplace introduces potential heterogeneity regarding formulation quality and dosing variability, which may influence adverse event reporting. Furthermore, while the concurrent timeframe (2022-2025) was used to reduce temporal bias, the near-identical reporting rates (0.60% vs. 0.62%) reinforce the likelihood of a class-wide safety profile rather than a drug-specific one, consistent with the Weber Effect observed in post-marketing surveillance [12].

Strengths and limitations

The primary strength of this study is the use of a concurrent study period, which mitigates the Weber Effect by analyzing both drugs during the same high-visibility timeframe [12]. However, several limitations inherent to spontaneous reporting systems must be acknowledged. First, FAERS data is subject to underreporting and lacks a denominator (total number of users), preventing the calculation of true incidence rates. Second, the “healthy user bias” may influence results; patients prescribed the newer, more expensive tirzepatide may differ socioeconomically or clinically from the general semaglutide population. Third, the analysis cannot account for unmeasured variables such as menopausal status, exact weight-loss trajectory, duration of therapy, or concomitant anticoagulant use, which may affect bleeding risk. Finally, the analysis did not differentiate between FDA-approved products and compounded formulations, which may introduce variability in safety signals.

## Conclusions

This analysis of post-marketing surveillance data did not detect a disproportionate reporting signal suggesting an increased risk of gynecological hemorrhagic events with tirzepatide compared to semaglutide. While these findings suggest a comparable safety profile, the high proportion of consumer-driven reporting for tirzepatide warrants cautious interpretation. Future prospective studies are needed to confirm these findings in controlled clinical settings, ideally controlling for menopausal status and concomitant hormone use.
